# Attenuation of Bone Formation through a Decrease in Osteoblasts in Mutant Mice Lacking the GM2/GD2 Synthase Gene

**DOI:** 10.3390/ijms23169044

**Published:** 2022-08-12

**Authors:** Eri Sasaki, Kazunori Hamamura, Yoshitaka Mishima, Koichi Furukawa, Mayu Nagao, Hanami Kato, Kosuke Hamajima, Takuma Sato, Ken Miyazawa, Shigemi Goto, Akifumi Togari

**Affiliations:** 1 Department of Pharmacology, School of Dentistry, Aichi Gakuin University, Nagoya 464-8650, Aichi, Japan; 2 Department of Orthodontics, School of Dentistry, Aichi Gakuin University, Nagoya 464-8651, Aichi, Japan; 3 Department of Biomedical Sciences, Chubu University College of Life and Health Sciences, Kasugai 487-8501, Aichi, Japan; 4 Department of Pediatric Dentistry, School of Dentistry, Aichi Gakuin University, Nagoya 464-8651, Aichi, Japan

**Keywords:** glycosphingolipids, GM2/GD2 synthase, osteoblasts, GD1a

## Abstract

The ganglioside GD1a has been reported to promote the differentiation of mesenchymal stem cells to osteoblasts in cell culture systems. However, the involvement of gangliosides, including GD1a, in bone formation in vivo remains unknown; therefore, we herein investigated their roles in GM2/GD2 synthase-knockout (GM2/GD2S KO) mice without GD1a. The femoral cancellous bone mass was analyzed using three-dimensional micro-computed tomography. A histomorphometric analysis of bone using hematoxylin and eosin (HE) and tartrate-resistant acid phosphatase was performed to examine bone formation and resorption, respectively. Calcein double labeling was also conducted to evaluate bone formation. Although no significant differences were observed in bone mass or resorption between GM2/GD2S KO mice and wild-type (WT) mice, analyses of the parameters of bone formation using HE staining and calcein double labeling revealed less bone formation in GM2/GD2S KO mice than in WT mice. These results suggest that gangliosides play roles in bone formation.

## 1. Introduction

Gangliosides, sialic-acid-containing glycosphingolipids, are expressed in various tissues and are considered to play important roles in the development and maintenance of organs and tissues as well as in the pathogenesis of disease [1,2,3,4,5,6,7]. Gangliosides have been shown to protect against inflammation and neurodegeneration through the suppression of complement systems [8]. They have also been implicated in the pathogenesis of Alzheimer’s, Parkinson’s, and Huntington diseases [5,9]. Furthermore, gangliosides have been reported to regulate the secretion of various hormones, such as insulin in pancreatic beta cells and leptin in adipose cells [10,11]. Gangliosides, including GD3 and GD2, are not only cancer-associated antigens but also promote malignant properties in melanoma, osteosarcoma, and breast cancer [12,13,14,15].

Previous studies demonstrated the involvement of gangliosides in bone metabolism [16,17,18,19]. The suppression of age-related bone resorption was detected in mutant mice lacking the GD3 synthase gene, which is essential for the production of b-series gangliosides [19]. Furthermore, the a-series ganglioside GD1a has been shown to play a role in the differentiation of mesenchymal stem cells (MSCs) to osteoblasts [16,17,18]. Although findings obtained using a cell culture system suggest that GD1a promotes bone formation, its contribution to bone formation in vivo remains unknown.

**Figure 1:**
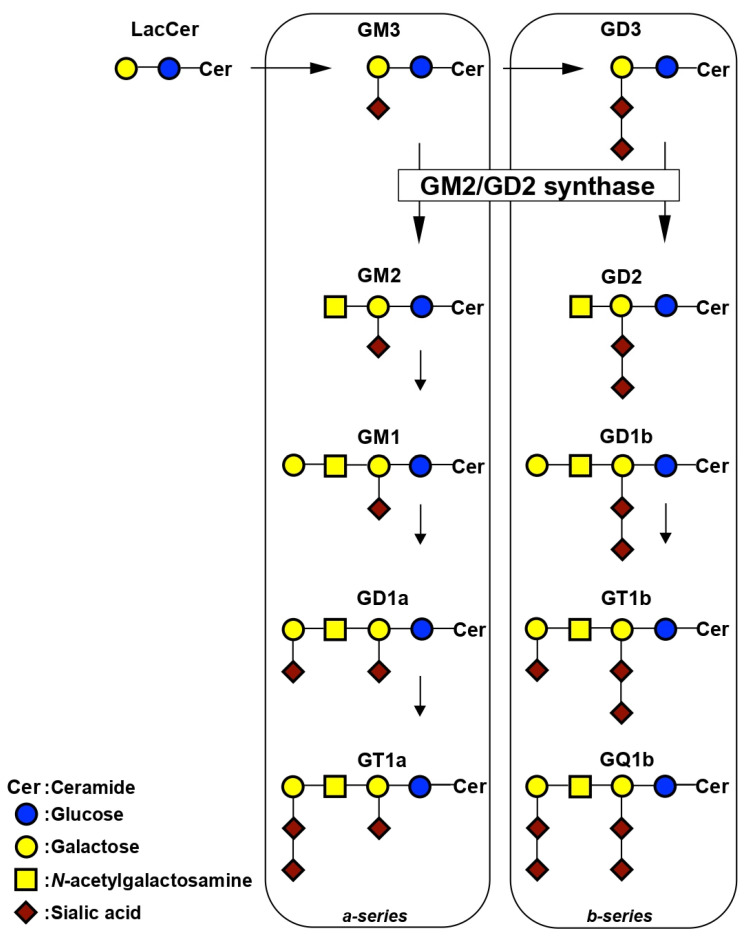
Synthetic pathway of gangliosides.

GM2/GD2 synthase is essential for the production of a-series (GM2, GM1, GD1a, and GT1a) and b-series (GD2, GD1b, GT1b, and GQ1b) gangliosides (Figure 1). The GM2/GD2 synthase, β1,4-*N*-acetylgalactosaminyltransferase, transfers *N*-acetylgalactosamine to GM3 and GD3, resulting in the production of GM2 and GD2, respectively. The roles of GM2/GD2 synthase in maintaining body homeostasis have been elucidated using GM2/GD2 synthase-knockout (GM2/GD2S KO) mice [20,21,22,23,24,25]. These mice are infertile due to a dysfunction in the transport of testosterone to the seminiferous tubes [21]. Previous studies demonstrated that the deletion of GM2/GD2 synthase in mice resulted in neurodegeneration and impaired neuroregeneration [22,23,24]. Furthermore, the lack of GM2/GD2 synthase reduced cognitive function and plasticity in the hippocampus [25]. However, the role of GM2/GD2 synthase in bone metabolism, such as bone formation and resorption, remains unknown.

Therefore, the present study examined the involvement of gangliosides, including GD1a, in bone formation by analyzing the femoral cancellous bone mass in wild-type (WT) and GM2/GD2S KO mice lacking GD1a, using three-dimensional micro-computed tomography (3D-μCT). A histomorphometric analysis of bone was performed using hematoxylin and eosin (HE) and tartrate-resistant acid phosphatase (TRAP) to assess bone formation and resorption, respectively. Calcein double labeling was also conducted to evaluate bone formation.

The results obtained herein demonstrate that bone formation was suppressed in GM2/GD2S KO mice through a decrease in the number of osteoblasts. The present study is the first to indicate the contribution of gangliosides to bone formation in vivo.

## 2. Results

### 2.1. Expression of Gangliosides in Osteoblasts

**Figure 2:**
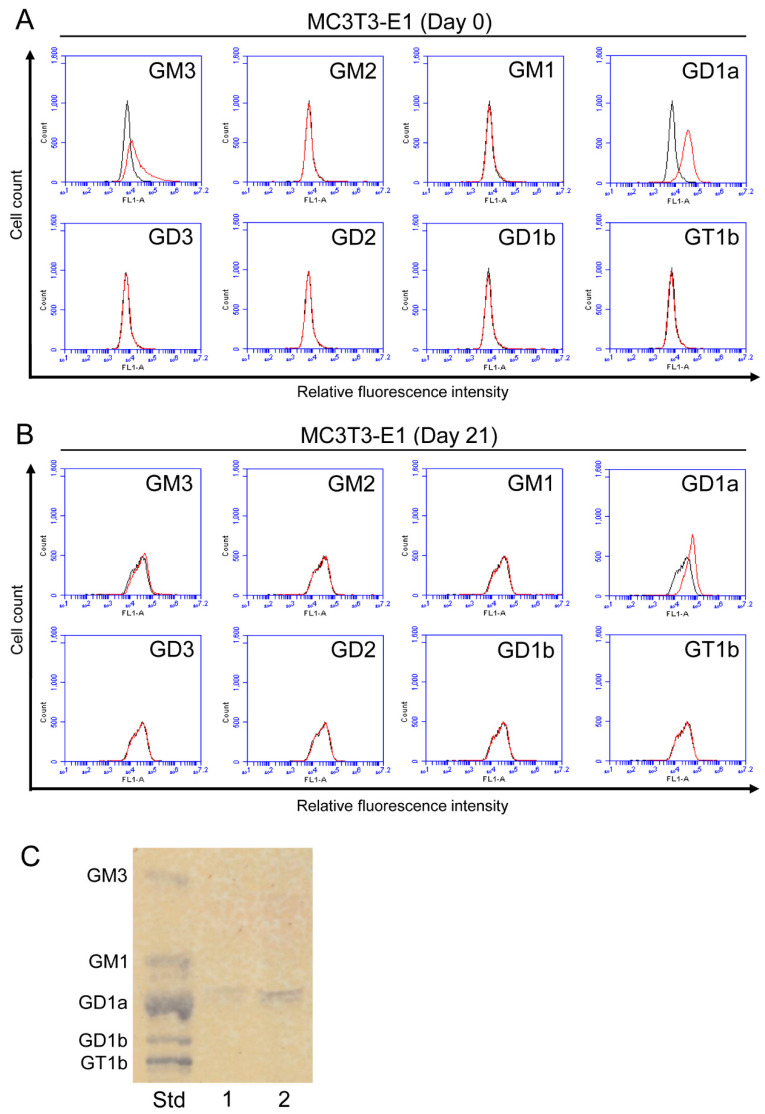
Expression of gangliosides in osteoblasts. (**A**) The expression levels of gangliosides (GM3, GM2, GM1, GD1a, GD3, GD2, GD1b, and GT1b) in MC3T3-E1 cells before the induction of osteoblastogenesis (day 0) were examined using a flow cytometric analysis. (**B**) The expression levels of gangliosides (GM3, GM2, GM1, GD1a, GD3, GD2, GD1b, and GT1b) in MC3T3-E1 cells on day 21 after the induction of osteoblastogenesis were examined using a flow cytometric analysis. Red line: anti-ganglioside monoclonal antibodies (mAbs) (+); gray line: anti-ganglioside mAbs (−). (**C**) The expression of gangliosides in MC3T3-E1 cells was analyzed by TLC. Acidic fractions derived from 21 (Lane 1) or 42 (Lane 2) mg cell pellets were applied in the individual lanes. Std: Standard.

A flow cytometric analysis was performed to assess the expression of a-series gangliosides (GM3, GM2, GM1, and GD1a) and b-series gangliosides (GD3, GD2, GD1b, and GT1b) in osteoblasts (Figure 2A,B). The expression of GM3 and GD1a was detected in MC3T3-E1 mouse osteoblast cells (Figure 2A). Decreases were observed in the expression levels of GM3 and GD1a after osteoblastogenesis had been induced (day 21) (Figure 2B). The expression of GM2, GM1, GD3, GD2, GD1b, and GT1b was negligible or absent in MC3T3-E1 cells. GD1a was also detected in TLC analysis (Figure 2C).

### 2.2. Expression of Gangliosides in RAW264.7 Mouse Pre-Osteoclast Cells with or without Receptor Activator of Nuclear Factor Kappa-B Ligand (RANKL)

**Figure 3:**
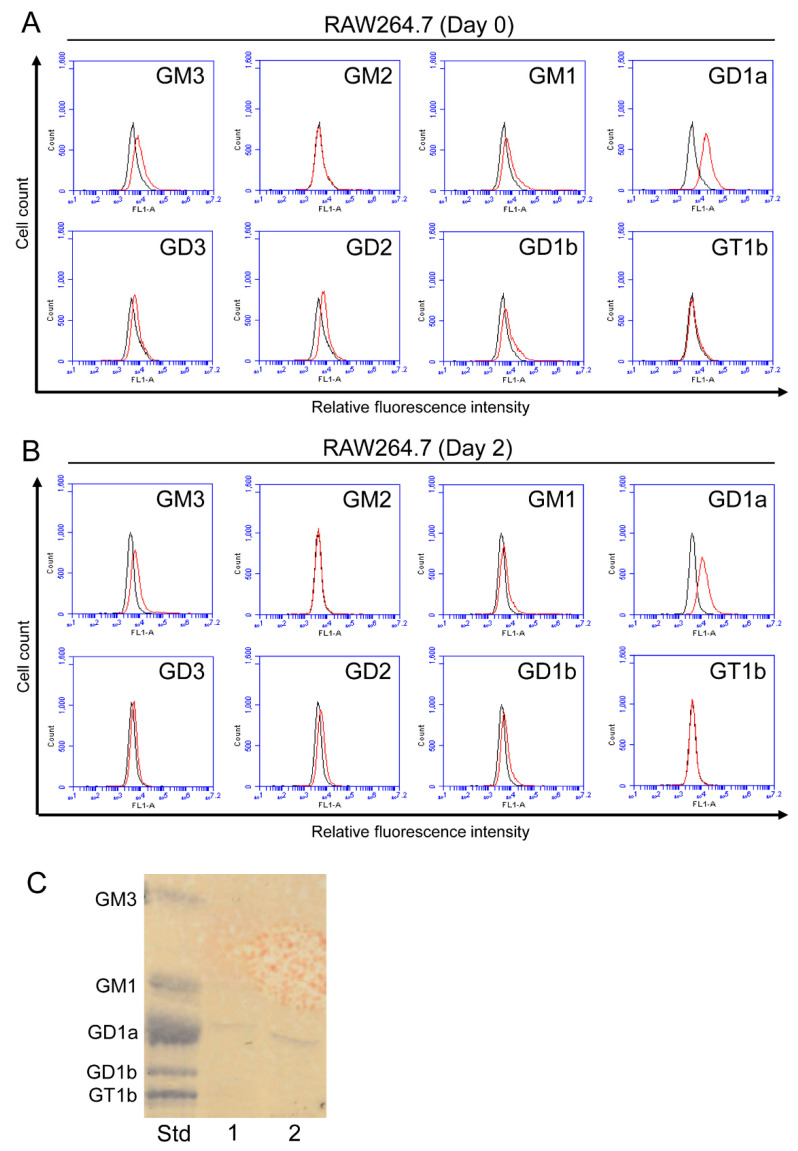
Expression of gangliosides in RAW264.7 cells with or without RANKL. (**A**) The expression levels of gangliosides (GM3, GM2, GM1, GD1a, GD3, GD2, GD1b, and GT1b) in RAW264.7 cells before the administration of RANKL (day 0) were measured using a flow cytometric analysis. (**B**) The expression levels of gangliosides (GM3, GM2, GM1, GD1a, GD3, GD2, GD1b, and GT1b) in RAW264.7 cells on day 2 after the administration of RANKL were examined using a flow cytometric analysis. Red line: anti-ganglioside monoclonal antibodies (mAbs) (+); gray line: anti-ganglioside mAbs (−). (**C**) The expression of gangliosides in RAW264.7 cells was analyzed by TLC. Acidic fractions derived from 21 (Lane 1) or 42 (Lane 2) mg cell pellets were applied in the individual lanes. Std: Standard.

The expression of GM3, GM1, GD1a, GD3, GD2, and GD1b was detected in RAW264.7 cells (Figure 3A). After RAW264.7 cells were induced to differentiate to osteoclasts (day 2), decreases were observed in the expression of these gangliosides (Figure 3B). The expression of GM2 and GT1b was negligible or absent in RAW264.7 cells. GD1a was also detected in TLC analysis (Figure 3C).

### 2.3. The Change in the Ganglioside Composition in Osteoblasts by Knockout or Knockdown of GM2/GD2 Synthase

**Figure 4:**
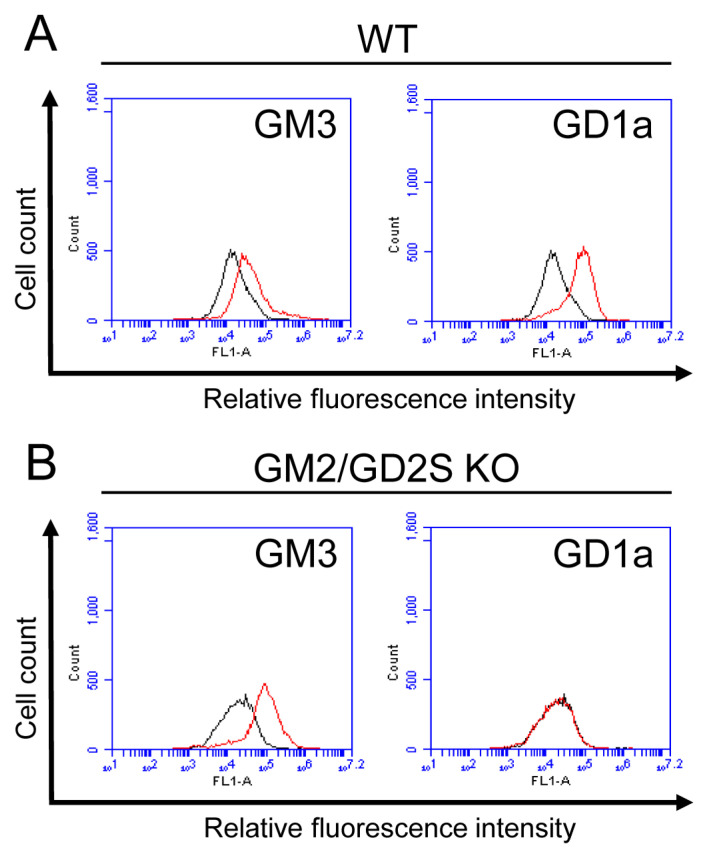
Expression of gangliosides in osteoblasts from WT mice and GM2/GD2S KO mice. (**A**) The expression levels of gangliosides (GM3 and GD1a) in osteoblasts from WT mice using a flow cytometric analysis. (**B**) The expression levels of gangliosides (GM3 and GD1a) in osteoblasts from GM2/GD2S KO mice using a flow cytometric analysis. Red line: anti-ganglioside monoclonal antibodies (mAbs) (+); gray line: anti-ganglioside mAbs (−).

**Figure 5:**
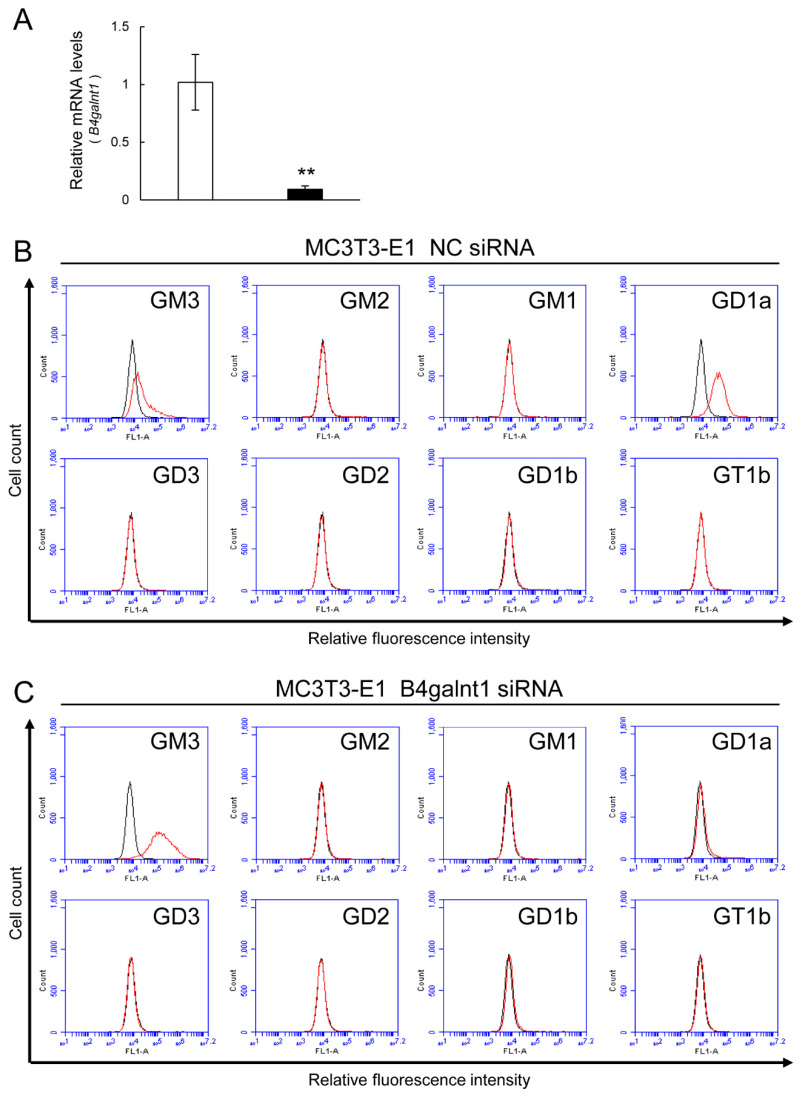
The change in the ganglioside composition in MC3T3-E1 cells by knockdown of GM2/GD2 synthase using siRNA. NC = negative control. (**A**) Reduction in *B4galnt1* by siRNA treatment. Results are shown as the mean ± S.D. Double asterisks indicate *p* < 0.01. The expression levels of gangliosides (GM3, GM2, GM1, GD1a, GD3, GD2, GD1b, and GT1b) in MC3T3-E1 cells after transfection of NC siRNA (**B**) or B4galnt1 siRNA (**C**) using a flow cytometric analysis. Red line: anti-ganglioside monoclonal antibodies (mAbs) (+); gray line: anti-ganglioside mAbs (−).

Osteoblasts from WT mice expressed GD1a. On the other hand, the osteoblasts from GM2/GD2S KO mice did not express GD1a at all (Figure 4). The expression of GM3 in the osteoblasts from GM2/GD2S KO mice was higher than that in the osteoblasts from WT mice. Furthermore, we analyzed the change in the ganglioside composition in MC3T3-E1 cells by knockdown of the GM2/GD2 synthase gene (*B4galnt1*) using the method of siRNA. *B4galnt1* was approximately reduced by 90% through its knockdown using siRNA (Figure 5A). GD1a was rarely expressed by knockdown of GM2/GD2 synthase. On the other hand, GM3 expression was elevated by knockdown of the GM2/GD2 synthase gene (Figure 5B). Collectively, knockout or knockdown of GM2/GD2 synthase revealed an elevation in GM3 instead of a reduction in GD1a.

### 2.4. The Change in the Ganglioside Composition in Pre-Osteoclasts by Knockout of GM2/GD2 Synthase

**Figure 6:**
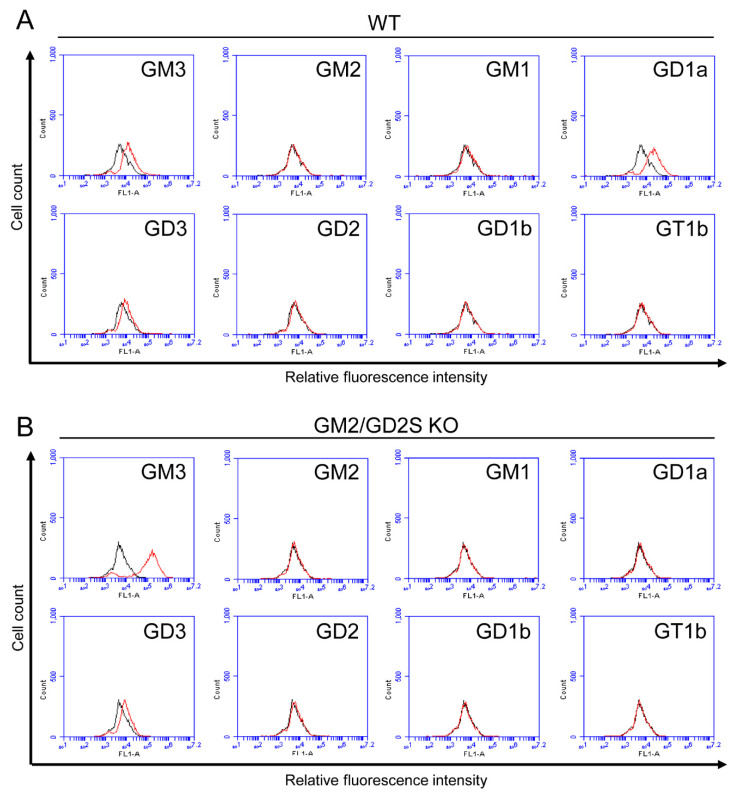
Expression of gangliosides in pre-osteoclasts from WT mice and GM2/GD2S KO mice. (**A**) The expression levels of gangliosides (GM3, GM2, GM1, GD1a, GD3, GD2, GD1b, and GT1b) in pre-osteoclasts from WT mice using a flow cytometric analysis. (**B**) The expression levels of gangliosides (GM3, GM2, GM1, GD1a, GD3, GD2, GD1b, and GT1b) in pre-osteoclasts from GM2/GD2S KO mice using a flow cytometric analysis. Red line: anti-ganglioside monoclonal antibodies (mAbs) (+); gray line: anti-ganglioside mAbs (−).

We analyzed the change in the ganglioside composition between pre-osteoclasts from WT and GM2/GD2S KO mice (Figure 6). Pre-osteoclasts from WT mice mainly expressed GM3, GD1a, and GD3 (Figure 6A). GD1a was not expressed by knockdown of GM2/GD2 synthase at all (Figure 6B). Similar to osteoblasts from GM2/GD2S KO mice, pre-osteoclasts from the KO mice also showed upregulation of GM3.

### 2.5. The Femoral Cancellous Bone Mass Did Not Differ between WT and GM2/GD2S KO Mice

**Figure 7:**
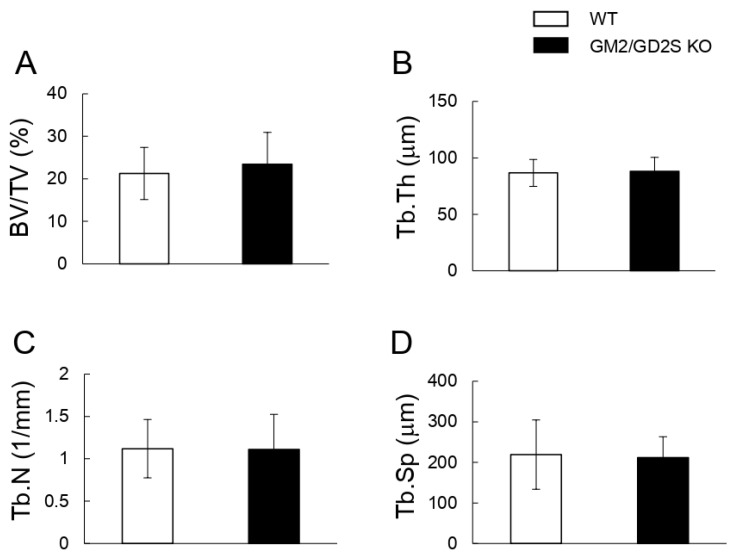
A GM2/GD2 synthase deficiency did not affect the femoral cancellous bone mass. Bone densitometric analysis of the distal region of the femur in WT and GM2/GD2S KO mice by 3D-μCT. Twelve-week-old mice (male, *n* = 15 for WT, *n* = 12 for GM2/GD2S KO). (**A**) Bone volume/total volume (BV/TV, %) is shown. (**B**) Trabecular thickness (Tb.Th, μm) is shown. (**C**) The trabecular number (Tb.N, 1/mm) is shown. (**D**) Trabecular separation (Tb.Sp, μm) is shown. Results are shown as the mean ± S.D.

To evaluate the involvement of GM2/GD2 synthase in bone volume, we analyzed bone volume/total volume (BV/TV) using μCT. No significant differences were observed in BV/TV, trabecular thickness (Tb.Th), trabecular number (Tb.N), or trabecular separation (Tb.Sp) between WT and GM2/GD2S KO mice (Figure 7).

### 2.6. Decreases in Parameters of Bone Formation by a Deficiency in GM2/GD2S Synthase

**Figure 8:**
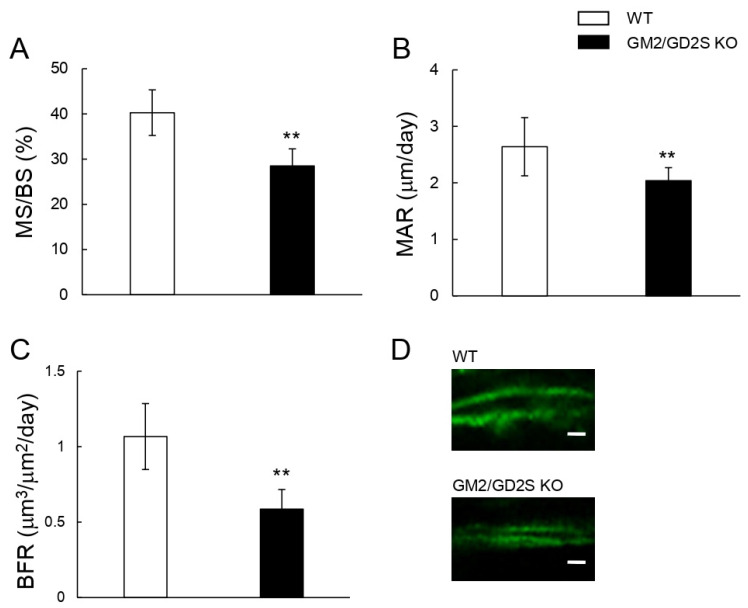
Bone formation is suppressed by a GM2/GD2 synthase deficiency. Fourteen-week-old mice (male, *n* = 9 for WT, *n* = 6 for GM2/GD2S KO). (**A**) The mineral surface/bone surface (MS/BS, %) is shown. (**B**) The mineral apposition rate (MAR, μm/day) is shown. (**C**) The bone formation rate (BFR, μm^3^/μm^2^/day) is shown. (**D**) Images of calcein double labeling of femoral cancellous bone. Scale bars are 5 μm. Results are shown as the mean ± S.D. Double asterisks indicate *p* < 0.01.

Calcein double labeling was performed to assess the bone formation rate. The mineral surface/bone surface (MS/BS), mineral apposition rate (MAR), and bone formation rate (BFR) were significantly lower in GM2/GD2S KO mice than in WT mice (Figure 8). MS/BS, MAR, and BFR were approximately reduced by 29%, 23%, and 45%, respectively. These results show that GM2/GD2 synthase was involved in bone formation.

### 2.7. Decreased Number of Osteoblasts in Femoral Cancellous Bone by a Deficiency in GM2/GD2S Synthase

**Figure 9:**
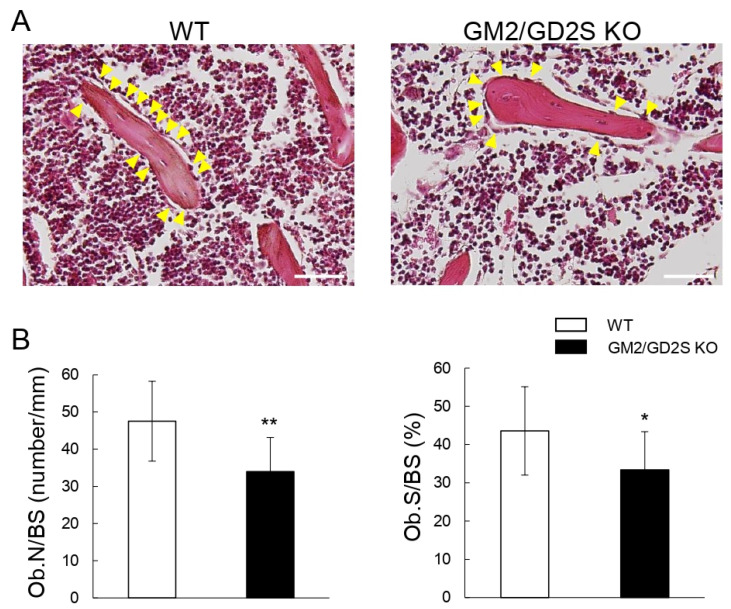
Decreases in the number of osteoblasts by a GM2/GD2 synthase deficiency. Twelve-week-old mice (male, *n* = 12 for WT, *n* = 12 for GM2/GD2S KO). (**A**) Images of hematoxylin and eosin (HE)-stained femoral cancellous bone. Images obtained for WT mice (**left**) and GM2/GD2S KO mice (**right**) are shown. Scale bars are 100 μm. Yellow arrowheads indicate osteoblasts. (**B**) The number of osteoblasts/bone surface (Ob.N/BS, number/mm) and osteoblast surface/bone surface (Ob.S/BS, %) are shown. Results are shown as the mean ± S.D. Single and double asterisks indicate *p* < 0.05 and *p* < 0.01, respectively.

The number of osteoblasts/bone surface (Ob.N/BS) and osteoblast surface/bone surface (Ob.S/BS) were significantly lower in GM2/GD2S KO mice than in WT mice (Figure 9). Ob.N/BS and Ob.S/BS were approximately reduced by 28% and 23%, respectively. These results may show that GM2/GD2 synthase affected the proliferation of osteoblasts.

### 2.8. The Number of Osteoclasts Did Not Significantly Differ between WT and GM2/GD2S KO Mice

**Figure 10:**
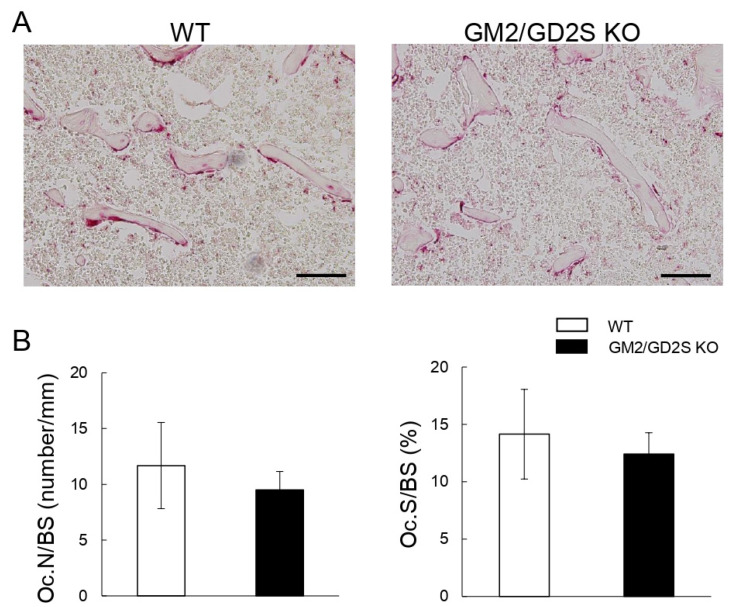
No significant effects of a GM2/GD2 synthase deficiency on bone resorption parameters. (**A**) Images of TRAP-stained femoral cancellous bone. Images obtained for WT mice (**left**) and GM2/GD2S KO mice (**right**) are shown. Scale bars are 100 μm. (**B**) The number of osteoclasts/bone surface (Oc.N/BS, number/mm) and osteoclast surface/bone surface (Oc.S/BS, %) are shown. Twelve-week-old mice (male, *n* = 13 for WT, *n* = 10 for GM2/GD2S KO). Results are shown as the mean ± S.D.

The parameters of bone resorption, including the number of osteoclasts/bone surface (Oc.N/BS) and osteoclast surface/bone surface (Oc.S/BS), were slightly lower in GM2/GD2S KO mice than in WT mice, but there were no significant differences (Figure 10). These results show that GM2/GD2 synthase did not affect bone resorption.

## 3. Discussion

The present results demonstrate the negative impact of a GM2/GD2 synthase deficiency in mice on bone formation via decreases in the number of osteoblasts. MC3T3-E1 osteoblast cells express GM3 and GD1a, while RAW264.7 macrophage cells express GM3, GM1, GD1a, GD3, GD2, and GD1b. Although bone mass did not significantly differ between WT and GM2/GD2S KO mice, a histomorphometric analysis of bone in addition to calcein double labeling showed that bone formation was significantly lower in GM2/GD2S KO mice than in WT mice. On the other hand, no significant differences were observed in bone resorption parameters between WT and GM2/GD2S KO mice.

Glycosphingolipids have been reported to play important roles in the proliferation and differentiation of osteoblasts [16,17,18,26,27]. Globo-series Gb4 is expressed in osteoblasts, and its deletion in mice negatively affected bone formation through a decrease in the number of osteoblasts [26]. Furthermore, the a-series ganglioside GD1a has been shown to promote the differentiation of MSCs into osteoblasts by promoting the phosphorylation of epidermal growth factor receptors [16,17]. Furthermore, the inhibition of glucosylceramide synthase, which is important for the production of all glycosphingolipids, reduced the expression levels of Gb4 and GD1a, thereby suppressing the proliferation of mouse osteoblasts [27].

As shown in Figure 2A, GM3 and GD1a were mainly expressed in osteoblasts. Furthermore, as shown in Figure 4 and Figure 5, knockout or knockdown of GM2/GD2 synthase resulted in the deletion or suppression of the expression level of GD1a, leading to an increase in the expression level of GM3. A previous study reported that the treatment of human MSCs with GD1a promoted their differentiation to osteoblasts [16]. On the other hand, the treatment of MSCs with GM3 suppressed their differentiation to osteoblasts [16]. Collectively, reductions in the number of osteoblasts in GM2/GD2S KO mice may be caused by the change in the ganglioside composition such as the deletion of GD1a and the increase in GM3. In order to clarify that, it will be necessary to examine whether the treatment of GD1a or GM3 in osteoblasts affects proliferation. Since GD1a was deleted in GM2/GD2S KO mice in the present study, reductions in the number of osteoblasts by the change in the ganglioside composition may have had a negative impact on bone formation, as shown in Figure 8 and Figure 9.

GD1a was expressed in immature osteoblast cells (MC3T3-E1 cells), and its expression level decreased in mature osteoblasts after the induction of osteoblastogenesis (Figure 2). This result indicates the importance of GD1a in regulating the proliferation of immature osteoblasts. In addition to previous findings showing that GD1a regulates the differentiation of MSCs to osteoblasts [16,17,18], it has been suggested to play a role in the proliferation of immature osteoblasts.

**Figure 11:**
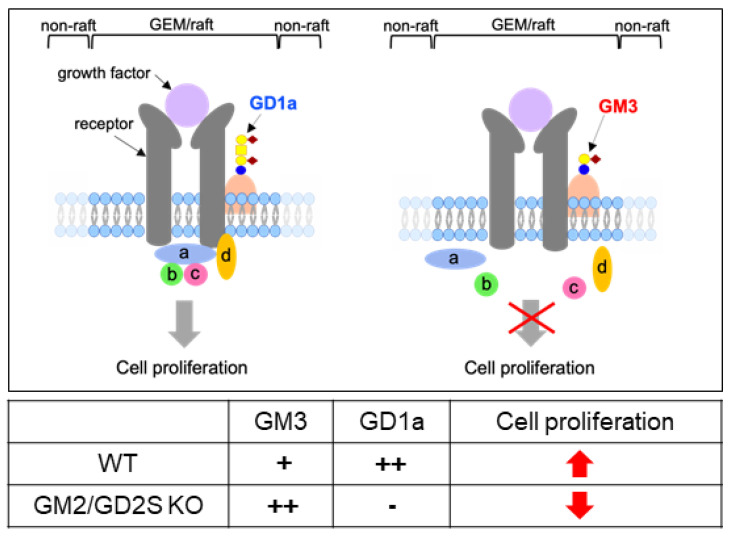
Schematic illustration of the proposed mechanism by which GD1a and GM3 regulate osteoblast proliferation. a, b, c, and d: signal transduction molecules.

Gangliosides are known to be enriched in the glycolipid-enriched microdomain (GEM)/rafts on the plasma membrane [28] and promote or suppress the cell signaling through the GEM/rafts [7,29,30,31,32]. GD1a and GM3 may interact with the growth factor receptors that regulate the cell proliferation in GEM/rafts, resulting in the enhancement or attenuation of the proliferation in osteoblasts. Here, we propose the mechanism by which GD1a and GM3 regulate osteoblast proliferation in GEM/rafts (Figure 11).

The mechanisms by which gangliosides contribute to bone formation were not elucidated in detail in the present study. Nevertheless, the results obtained clearly demonstrate, for the first time, that gangliosides regulate bone formation in vivo.

## 4. Materials and Methods

### 4.1. Mice

GM2/GD2S KO mice were generated as previously reported [20]. WT and GM2/GD2S KO mice were mated to yield heterozygotes, which were then mated, and the genotypes of the offspring were screened according to a previously reported method [20]. Mice were housed in cages with free access to water and food under a 12 h light/dark cycle. Protocols in the present study received approval from the Aichi Gakuin University Animal Research Committee (approval number AGUD312; 29 October 2015, AGUD463; 28 May 2020) (Nagoya, Japan) and were performed in accordance with the Guidelines for Animal Experiments of Aichi Gakuin University. Seventeen WT mice and sixteen GM2/GD2S KO mice were used for 3D-μCT, HE, and TRAP. Nine WT mice and six GM2/GD2S KO mice were used for calcein double labeling. Four WT mice and five GM2/GD2S KO mice were used for in vitro experiments.

### 4.2. Cell Culture

Cultures of MC3T3-E1 cells established from newborn mouse calvaria [33] and RAW264.7 (monocyte/macrophage) cells from mouse ascites [34] in α-Minimum Essential Medium (Wako Pure Chemical Industries, Ltd., Osaka, Japan) supplemented with 10% fetal bovine serum (Sigma-Aldrich, St. Louis, MO, USA) and antibiotics (100 U/mL penicillin, 100 μg/mL streptomycin; Wako Pure Chemical Industries, Ltd.) were performed at 37 °C with 5% CO_2_ in an incubator with a humidified environment.

### 4.3. Induction of Primary Osteoblasts

Mouse bone marrow cells from WT and GM2/GD2S KO mice were plated in 10 cm dishes and cultured in α-Minimum Essential Medium supplemented with 15% fetal bovine serum, 50 μg/mL ascorbic acid (Wako Pure Chemical Industries, Ltd.), 10^−8^ M dexamethasone (Sigma-Aldrich), 10 mM β-glycerophosphate (Sigma-Aldrich), and antibiotics (100 U/mL penicillin, 100 μg/mL streptomycin) [35]. Cells were subjected to a flow cytometric analysis after 6 days in culture.

### 4.4. Culture of Primary Pre-Osteoclasts

Mouse bone marrow cells from WT and GM2/GD2S KO mice were plated in 150 mm dishes and cultured with 10 ng/mL macrophage colony-stimulating factor (M-CSF, PeproTech, Inc., Rocky Hill, NJ, USA) for 3 days. The surface-attached cells were used for a flow cytometric analysis [36].

### 4.5. Antibodies

The anti-GD1a monoclonal antibody (mAb) D266 [37], anti-GD2 mAb 220-51 [29], anti-GD1b mAb 370 [29,38], and anti-GT1b mAb 549 [29] were produced in Dr. Furukawa’s laboratory. Dr. Lloyd J. Old at the Memorial Sloan-Kettering Cancer Center provided anti-GM2 mAb 10-11 [39] and anti-GD3 mAb R24 [29,40,41]. Anti-GM3 mAb GMR6 and anti-GM1 mAb GMB16 were obtained from Tokyo Chemical Industry Co., Ltd. (Tokyo, Japan) and FITC-conjugated anti-mouse IgG and anti-mouse IgM from Affymetrix eBioscience (San Diego, CA, USA).

### 4.6. Differentiation to Mature Osteoblasts

MC3T3-E1 cells were grown to confluence in 50 μg/mL ascorbic acid and 5 mM β-glycerophosphate. The culture medium was replaced every second day. Cells were subjected to a flow cytometric analysis after 21 days in culture.

### 4.7. Induction of Osteoclastogenesis

RAW264.7 cells were plated in 150 mm culture dishes and treated with 50 ng/mL RANKL (PeproTech, Inc.). After 2 days, cells were subjected to a flow cytometric analysis.

### 4.8. Flow Cytometry

An Accuri^TM^ C6 Flow Cytometer (BD Biosciences, San Jose, CA, USA) was employed to assess the cell surface expression of gangliosides with the following anti-ganglioside mAbs: anti-GM3 mAb (mouse IgM, GMR6) (1:100), anti-GM2 mAb (mouse IgM, 10-11) (1:100), anti-GM1 mAb (mouse IgM, GMB16) (1:67), anti-GD1a mAb (mouse IgM, D266) (1:100), anti-GD3 mAb (mouse IgG, R24) (1:100), anti-GD2 mAb (mouse IgG, 220-51) (1:100), anti-GD1b mAb (mouse IgM, 370) (1:100), and anti-GT1b mAb (mouse IgM, 549) (1:100). Cells were incubated on ice for 60 min with each mAb and then stained on ice for 45 min using FITC-conjugated anti-mouse IgG (1:200) or IgM (1:200). Control cells for the flow cytometric analysis were incubated with the secondary antibody only [26]. Of note, for RAW264.7 cells and pre-osteoclasts (macrophages), TruStain FcX PLUS (anti-mouse CD16/32) antibody was used for blocking non-specific binding of immunoglobulin to the Fc receptors (BioLegend, San Diego, CA, USA) before incubation of primary antibodies.

### 4.9. Extraction of Glycolipids

Glycolipids were extracted with chloroform/methanol (2:1, *v*/*v*), (1:1), and (1:2), sequentially. The extracts were pooled and dried, and acidic fractions were isolated using DEAE Sephadex A-50^TM^ (Cytiva, Uppsala, Sweden) ion exchange column chromatography after desalting with a Sep-Pak C18 Cartridge^TM^ (Waters, Milford, MA, USA). The acidic fractions were dissolved in C:M (1:1, *v*/*v*) and applied to thin-layer chromatography (TLC).

### 4.10. TLC

The acidic fractions were dissolved in chloroform/methanol (1:1, *v*/*v*), and fractions equivalent to 570 mg (MC3T3-E1 cells) and 250 mg (RAW264.7 cells) of cell pellets were applied to a silica-gel-coated plate (HPTLC Silica gel 60^TM^, Merck, Darmstadt, Germany). TLC of acidic fractions was performed using a solvent system, chloroform/methanol/0.2% CaCl_2_ (55:45:10), and bands were detected by resorcinol spray. As a standard, a ganglioside mixture from bovine brain gangliosides (Calbiochem, San Diego, CA, USA) plus GM3 was used.

### 4.11. Quantitative Real-Time PCR (qPCR)

An RNeasy Plus mini kit (Qiagen, Germantown, MD, USA) was used to extract total RNA. A high-capacity cDNA reverse transcription kit (Applied Biosystems, Carlsbad, CA, USA) was employed for reverse transcription, and TakaRa Thermal Cycler Dice Real Time System III with THUNDERBIRD SYBR qPCR mix kits (TOYOBO, Osaka, Japan) for qPCR. Total RNA was used as the template for reverse transcription under the following cycling conditions: at 25 °C for 10 min, 37 °C for 120 min, and 85 °C for 5 min. PCR cycling parameters were an initial hold at 95 °C for 10 min, followed by 40 cycles at 95 °C for 15 s, and 60 °C for 1 min. The *B4galnt1* (GM2/GD2 synthase gene) (forward: 5′-GCTGGGTCTCCTGTACTCCA-3′, and backward: 5′-TCCTCCCTTGGATTCACAAC-3′) mRNA level was then measured with *Gapdh* (forward: 5′-TGCACCACCAACTGCTTAG-3′, and backward: 5′-GGATGCAGGGATGATGTTC-3′) as the internal control.

### 4.12. Knockdown of B4galnt1 (GM2/GD2 Synthase Gene) by siRNA

MC3T3-E1 cells were treated with siRNA specific to B4galnt1 (5′-GACUUUUCUUCGUUAUGAU-3′; Life Technologies, Carlsbad, CA, USA). As a non-specific control, a negative control siRNA (Silencer Select Negative Control #1; Life Technologies) was used. Cells were transiently transfected with siRNA in Opti-MEM I medium with Lipofectamine RNAiMAX (Life Technologies). The efficiency of silencing was assessed with qPCR 48 h after transfection. The cells at 96 h after transfection were used for a flow cytometric analysis.

### 4.13. Three-Dimensional Micro-Computed Tomography Analysis

Three-dimensional micro-computed tomography (CosmoScan R-mCT-GX-T1; RIGAKU, Tokyo, Japan) scanning was initiated 0.5 mm above the distal femoral growth plate, and cancellous bone parameters in 75 consecutive 20 μm-thick sections were assessed using TRI/3D-BON (Ratoc, Tokyo, Japan) software according to a previously reported method [19].

### 4.14. Calcein Double Labeling to Measure the Bone Formation Rate

MS/BS, MAR, and BFR were assessed using calcein double labeling. Mice were intraperitoneally administered calcein (8 μg/g; Sigma, St. Louis, MO, USA) 3 days and 1 day before being euthanized. Following their fixation in 4% paraformaldehyde (PFA), femurs were cut to obtain undecalcified 5 μm-thick sections. A bone fraction with a rectangular area of 0.34 mm^2^ (0.5 × 0.67 mm), with the closest and furthest edges being 0.5 and 1.0 mm medial to the growth plate, respectively [26], was obtained from metaphyseal cancellous bone in the femur and used to measure MS/BS, MAR, and BFR.

### 4.15. TRAP Staining

TRAP staining was conducted according to a previously reported method [42]. Briefly, slides of femur samples were subjected to TRAP staining at 37 °C for 60 min using sodium acetate buffer (0.1 M, pH 5.0) with naphthol AS-MX phosphate, Fast Red Violet LB Salt, and MnCl_2_ in the presence of sodium tartrate.

### 4.16. Histomorphometric Analysis of Bone

Femur samples fixed in 4% PFA were decalcified in 10% ethylenediaminetetraacetic acid (EDTA) for three weeks and embedded in paraffin. Samples were then sectioned at a thickness of 5 μm and subjected to HE and TRAP staining. HE-stained slides were used to assess Ob.N/BS and Ob.S/BS according to a previously reported method [19]. TRAP-stained slides were used to assess Oc.N/BS and Oc.S/BS [19]. Parameters were measured within an area of 0.8 mm^2^ (1.0 × 0.8 mm), with the closest and furthest edges being 2.0 and 3.0 mm medial to the growth plate in the proximal ends of the femur, respectively [43].

### 4.17. Statistical Analysis

Results are shown as the mean ± S.D. The significance of differences was examined using Student’s *t*-test at *p* < 0.05, with single and double asterisks indicating *p* < 0.05 and *p* < 0.01, respectively.

## 5. Conclusions

The present study showed that a GM2/GD2 synthase deficiency in mice had a negative impact on bone formation by decreasing the number of osteoblasts.

## Data Availability

The data presented in this study will be available on reasonable request to the corresponding author.
